# Dissecting Motor and Cognitive Component Processes of a Finger-Tapping Task With Hybrid Dopamine Positron Emission Tomography and Functional Magnetic Resonance Imaging

**DOI:** 10.3389/fnhum.2021.733091

**Published:** 2021-11-29

**Authors:** Filip Grill, Jarkko Johansson, Jan Axelsson, Patrik Brynolfsson, Lars Nyberg, Anna Rieckmann

**Affiliations:** ^1^Department of Radiation Sciences, Umeå University, Umeå, Sweden; ^2^Umeå Center for Functional Brain Imaging, Umeå University, Umeå, Sweden; ^3^Department of Integrative Medical Biology, Umeå University, Umeå, Sweden; ^4^The Munich Center for the Economics of Aging, Max-Planck-Institute for Social Law and Social Policy, Munich, Germany

**Keywords:** finger tapping, PET, fMRI, dopamine, cognitive component, striatum

## Abstract

Striatal dopamine is involved in facilitation of motor action as well as various cognitive and emotional functions. Positron emission tomography (PET) is the primary imaging method used to investigate dopamine function in humans. Previous PET studies have shown striatal dopamine release during simple finger tapping in both the putamen and the caudate. It is likely that dopamine release in the putamen is related to motor processes while dopamine release in the caudate could signal sustained cognitive component processes of the task, but the poor temporal resolution of PET has hindered firm conclusions. In this study we simultaneously collected [11C]Raclopride PET and functional Magnetic Resonance Imaging (fMRI) data while participants performed finger tapping, with fMRI being able to isolate activations related to individual tapping events. The results revealed fMRI-PET overlap in the bilateral putamen, which is consistent with a motor component process. Selective PET responses in the caudate, ventral striatum, and right posterior putamen, were also observed but did not overlap with fMRI responses to tapping events, suggesting that these reflect non-motor component processes of finger tapping. Our findings suggest an interplay between motor and non-motor-related dopamine release during simple finger tapping and illustrate the potential of hybrid PET-fMRI in revealing distinct component processes of cognitive functions.

## Introduction

Simple motor tasks like finger tapping have frequently been used to probe the human motor system both in health (Riecker et al., 2003; Turesky et al., 2018) and disease (Elsinger et al., 2003; Wu et al., 2010; Wurster et al., 2015). The striatum is involved in the facilitation of desirable movements and inhibition of undesirable movements; striatal dopamine (DA) release patterns mediate the execution of desirable movements (Albin et al., 1989; DeLong, 1990; Calabresi et al., 2014). Additionally, the striatum supports the processing of information related to both higher cognitive functions (Cohen and Frank, 2009) and incentives (e.g., Haber and Knutson, 2010). The striatum receives projections from most of the cerebral cortex as well as DAergic input from the midbrain (Haber and Knutson, 2010), which makes the striatum a convergent area where DA modulates limbic, associative and sensorimotor functions.

Positron emission tomography (PET) is the primary imaging method to investigate DAergic functions in humans. [11C]Raclopride is an antagonist for the DA D2 receptors (Farde et al., 1986), and the binding profile of [11C]Raclopride has been shown sensitive to competition with endogenous DA (Laruelle, 2000). Binding competition occurs when endogenous DA levels are increased in the striatum, reducing the concentration of free D2 receptors available for [11C]Raclopride binding (Dewey et al., 1993). Using [11C]Raclopride and the “binding competition” principle, DA release in the bilateral putamen and the caudate during unrewarded finger tapping has been demontrated (Badgaiyan et al., 2003; Goerendt et al., 2003). Both Badgaiyan et al. (2003) and Goerendt et al. (2003) speculated that DA release in the putamen was reflective of motor demands, consistent with known anatomical projections to the motor cortex, while the caudate responses may have reflected non-motor processes such as learning or attention, which are likely to occur at different timescales than the transient motor specific aspects of the task. Indeed, [11C]Raclopride displacement studies have revealed DA release in the caudate and putamen during executive processes (Monchi et al., 2006; Dahlin et al., 2008; Lappin et al., 2009) and in the ventral striatum (VS) during rewarded conditions (Pappata et al., 2002; Joutsa et al., 2012; Jonasson et al., 2014). Thus, comparisons across studies support the hypothesis that DA release in striatal regions during motor tasks is reflective not only of the motor demands *per se* but also cognitive contributions. By this view, the striatum emerges as an important locus for the interplay between cognition and motor control. With the DA system playing a key role in many psychiatric and neurological disorders (Brisch et al., 2014; Belujon and Grace, 2017; Martini et al., 2018), a precise understanding of spatiotemporally specific DAergic functions in the human striatum is important. Several lines of work indicate a regionally distinctive functional architecture of striatal DA (Haber and Knutson, 2010), but direct evidence for such distinctions in humans has remained elusive. This omission primarily pertains to the inherently limited temporal resolution of *in vivo* PET-techniques (at the timescale of minutes at best), inhibiting the separation between transient motor activity and sustained cognitive component processes.

Recent technological developments have allowed the simultaneous acquisition of PET and fMRI. This opens up the possibility to investigate neurochemical processes such as DA release from PET concomitant with neurovascular responses [i.e., the blood-oxygen-level-dependent (BOLD) response from fMRI] during tasks in humans. Using the BOLD response, it is possible to investigate brain activity non-invasively at a timescale of seconds, which provides an opportunity to disentangle short periods of task states (i.e., finger movements) from sustained task set (e.g., related to attention or motivation; for review see Petersen and Dubis, 2012). In a simple finger tapping task, modeling the fMRI data as periods of movement vs. rest yields a robust BOLD response mostly confined to the putamen (Lehéricy et al., 2006; Witt et al., 2008).

In this study, we simultaneously collected [11C]Raclopride PET and fMRI to investigate DA release and BOLD response in nine human participants while they performed a finger tapping task consisting of long (several minutes) blocks of tapping and blocks of rest. Importantly, the task design permitted us to capture neuronal activation specific to the transient component of finger movements at the timescale of seconds using fMRI, while PET was used to identify striatal regions where DA release was related to the task at both faster and slower temporal scales. That is, the fMRI analysis was tuned to identify the regions that were more likely related to the fast component of motor activity, while the maps identified by using PET provided an overall spatial DAergic activity pattern regardless of task component process. By comparing the statistical spatial maps from both modalities, we hence theorized that signal overlap would reflect striatal DA release in response to the transient motor components of task, while DA release without task-specific BOLD response was hypothesized to reflect non-motor components of the task, e.g., motivation or attention. The aim of this study was to test the hypothesis that striatal DA release during motor function is associated with motor as well as non-motor processes, in a spatially distinct manner. In order to further understand the nature of non-motor contributions, we used the non-overlapping areas as seeds in a resting state functional connectivity analysis to map their functional coupling to cortical systems, thereby constraining the interpretation of their functional contribution.

## Materials and Methods

### Participants

Participants were recruited via ads placed around Umeå University campus, targeting young healthy adults between 20–40 years of age. Exclusion criteria included history of head trauma, current or past diagnosis of neurological or psychiatric illness, drug or alcohol dependence, and use of psychopharmaceuticals or stimulants other than caffeine or nicotine for the past 6 months. Individuals with MR-incompatible metallic implants or objects in their body were excluded. Pregnant or breast-feeding women, as well as individuals having previously undergone PET scanning for research purposes were excluded due to radiation safety reasons. All included participants were right-handed. One participant was excluded due to excessive head motion during the scan. The resulting sample size consisted of 9 healthy young adults (mean age = 24.9, *SD* = 4.2, range 20–34; mean height = 172.7, *SD* = 12.9, range 151–196; mean weight = 71.78, *SD* = 13.1, range 55–98; 5 females). This study was approved by the Regional Ethics Committee at Umeå University (2015/239-31).

### Procedure

Upon arrival, participants were informed about the study and signed an informed consent form. An intravenous needle used for infusion was placed in the left arm. Participants were then placed into the scanner bore where a mirror mounted on the scanner coil directed their gaze toward a screen located behind the scanner. Task instructions were prompted on the screen during the experiment. A T1-weighted structural image was collected, followed by a resting state T2^∗^-weighted sequence. Participants were injected with [11C]Raclopride at the start of the PET scan. Twenty minutes later the task T2^∗^-weighted sequence started.

### Experimental Design

**FIGURE 1:**
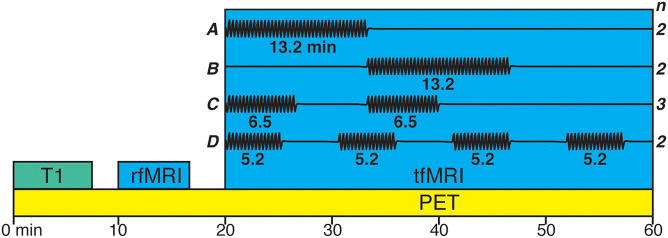
Variations of the finger tapping task paradigm. Black line in **(A–D)** represents blocks of tapping. Two participants had a single block (13.2 min; **A**) with the onset 20 min from PET acquisition start. Two participants had a single block (13.2 min; **B**) with the onset 33.33 min from PET acquisition start. Three participants had two blocks (6.5 min; **C**) with the onset 20 and 33.33 min from PET acquisition start. Two participants had four blocks (5.2 min; **D**) with the onset 20, 30.66, 41.33, and 52 min from PET acquisition start.

The task consisted of sequential finger tapping with the right hand (index, middle, ring, little finger). Participants were told to self-pace their tapping and were visually cued to tap for 10 s and then rest for 10 s until they were cued to start tapping again. The embedding of this on-off design within the task periods was chosen to fit the temporal resolution of BOLD response so that the BOLD signal would increase during the tapping event while allowing it to decrease during the rest inter trial intervals. The tapping task was partitioned into blocks with differing number of blocks, duration, and onset times between participants which can be seen in Figure 1. Note that, since the individual displacement maps were averaged across subjects for the main analysis, the individual differences in design were not of interest to the current study. Nevertheless, since it is unknown how short task blocks may be in [11C]Raclopride displacement study, we present the analyses of individual maps in Supplementary Material. These analyses showed that all task block lengths between 5 and 13 min were able to elicit displacement.

### PET/MRI Acquisition, Processing, and Analysis

Imaging was performed on a 3T General Electric Signa PET-MR system with a 15-channel head coil. Behavioral data (button presses) were recorded with an MR-compatible 4-button response pad from Cambridge Research Systems. The data was collected between November 2015 and March 2016.

#### T1-Weighted

Structural T1-weighted images were acquired for 7.36 min with the following acquisition parameters: [FOV: 25 × 20 cm^2^, matrix: 256 × 256, Slice Thickness: 1 mm, Slices: 180, TE: 3.1 ms, TR: 7,200 ms, Flip Angle: 12, Bandwidth: 244.1 Hz/Pixel]. T1 images were used for segmenting the brain into anatomical compartments using Freesurfer (Fischl, 2012) and normalization to standard MNI space using a preliminary 12 degrees of freedom registration with FMRIB’s Linear Image Registration Tool (FLIRT) followed by a non-linear registration using FMRIB’s Non-linear Image Registration Tool (FNIRT), resulting in 2 mm isotropic voxels.

#### Positron Emission Tomography

The participants were injected with a bolus plus constant infusion of [11C]Raclopride (Kbol = 105 min, Watabe et al., 2000) commencing at start of PET scan. Following the local standard protocol for [11C]Raclopride studies (e.g., Jonasson et al., 2014; Nevalainen et al., 2015), 250 MBq was delivered to the participant during the experiment. A 60 min (20 × 60 s, 30 × 80 s) dynamic time-of-flight acquisition and an MR-based attenuation correction was collected. The data was reconstructed to a voxel size of 1.56 × 1.56 × 2.78 mm^3^, employing a resolution recovery OSEM algorithm (3 iterations, 28 subsets, 3.0 mm post filter), with decay, randoms, scatter, and attenuation corrections applied. The data were then motion corrected using FSL’s mcflirt with mutual information as cost function to the 25th frame using framewise rigid body alignment, processed using a HYPR filter (Christian et al., 2010), and temporally smoothed using a three-frame Gaussian kernel ([0.25 0.50 0.25]).

Linear parametric neurotransmitter PET (lp-ntPET) was used to estimate voxelwise dynamic binding potentials (Normandin et al., 2012; Sander et al., 2013; Johansson et al., 2019). First, multilinear reference tissue modeling with fixed *k2’* was conducted. An additional time-dependent term was then fitted to the data for each task block to account for [11C]Raclopride displacement. The time-dependent term was defined as the best least-squares solution of a library of gamma functions (Madsen, 1992) with varying α controlling growth and decay rate. This approach takes into account inter-individual as well as inter-regional differences in [11C]Raclopride displacement, adaptive with the unknown shape and onset of dopamine release related to finger tapping. The best solution for each voxel results in individual t-statistics maps of [11C]Raclopride displacement during the tapping task.

Individual task timings were considered only in the first-level model. The individual *t* statistics maps were then taken to a second level analysis using FSL’s randomize function (512 permutations; uncorrected *p*-value) which estimated the group mean [11C]Raclopride displacement using a one-sample *t*-test, independent of each person’s individual on- and offsets (which were not of interest to the current analysis). Thus, the final group map provided a statistical map of coherent spatial locations of DA release during task as compared to rest.

#### Functional Magnetic Resonance Imaging

All BOLD fMRI data were collected with the same sequence parameters (FOV: 25.6, Matrix: 96 × 96, Slice Thickness: 3.6 mm, TE: 30 ms, TR: 4,000 ms, Flip Angle: 80°, Acceleration Factor: 2.0). Acquisition of BOLD resting state data started after the T1-weighted image and data was collected for 6.67 min. Acquisition of BOLD task data commenced 20 min after the PET acquisition started and was collected for 40 min (Figure 1).

The task data was pre-processed following conventional steps for fMRI as implemented in FSL FEAT^1^. Briefly, this included motion correction by volume-wise rigid body transformation to the first volume, slice timing correction, spatial smoothing (FWHM 5 mm), high pass (50 s) temporal filtering. Single subject task data was analyzed using a general linear model (GLM) with a single on-off regressor of interest describing finger tapping events. The beta estimates from the single subject analysis were taken into a second level group analysis using FSL’s randomize function (512 permutations; uncorrected *p*-value) estimating the group mean using a one-sample *t*-test.

The resting state data was motion corrected by volume-wise rigid body transformation to the first volume, slice timing corrected, and 24 motion parameters as well as framewise displacement outliers were regressed out from the data. Minimally preprocessed images were then non-linearly registered to MNI-space using FNIRT. White matter, cerebrospinal fluid, and global signal was regressed out and the data was spatially smoothed (FWHM 5 mm) and band pass filtered (high pass 0.01 Hz, low pass 0.1 Hz). Striatal seeds identified from the lp-ntPET analysis were used in a whole brain functional connectivity analysis to provide indications of their connected cortical targets and thereby constrain the interpretation of their functional contribution. For this, each time-series from the striatal seeds were individually correlated (Pearson’s correlation) with each voxel’s time-series for the whole brain. Individual correlation maps were then Fisher’s r-to-z transformed and entered to a second level group analysis. The resulting group t-statistic maps were given a threshold of *t* > 2.9 corresponding to an uncorrected *p*-value (one-tailed; df = 8) of 0.01 to investigate each seed’s strongest functional coupling. The group t-statistics maps were projected to a cortical surface for visualization purposes.

#### Modality Overlap

A first pass qualitative assessment of overlap/non-overlap between modalities was performed at an (arbitrary) t-threshold of 2.9 (*p* = 0.01) for the fMRI group map and a p-threshold of 0.05 for the group PET map. To ensure robustness of assessment a voxel overlap percentage count was conducted for stepwise [*t*(step) = 0.1] increasing t-thresholds [*t*(min) = 1, *t*(max) = 3.5]. This analysis was made to confirm that overlap/non-overlap definitions were threshold-independent. Overlapping and non-overlapping clusters were then assigned to their appropriate anatomical compartment (putamen, caudate, VS). Once overlap/non-overlap and anatomical compartment ROIs were established, the lp-ntPET analysis was performed again on the time activity curves extracted from the ROIs to confirm [11C]Raclopride displacement for each ROI and individual (Supplementary Material).

## Results

### Widespread Dopamine Release in Response to Finger Tapping

Mean finger tapping frequency during the task blocks was 1.95 ± 0.11 Hz. The voxelwise lp-ntPET analysis showed [11C]Raclopride displacement in several areas of the striatum. Four clusters were observed in the bilateral putamen, three clusters in the bilateral caudate, and two clusters in the bilateral VS (Table 1). Because of close spatial proximity of these smaller clusters within anatomically defined regions such as the caudate, the clusters within each anatomically defined region were combined for further analysis (Table 1).

**TABLE 1:** Group-level significant [11C]Raclopride displacement clusters.

	**Location**	**Number of voxels**	**Mean t-stat**	**Peak t-stat**	**Overlap**
Putamen	Left middle-anterior	78	2.63	6.19	YES
	Right anterior	28	2.37	3.26	YES
	Right middle	24	2.90	5.38	YES
	Right posterior	63	3.07	6.07	NO
Caudate	Left anterior	19	2.36	2.87	NO
	Right anterior	106	2.70	7.80	NO
	Right posterior	24	2.63	3.49	NO
VS	Left	27	2.36	3.34	NO
	Right	16	2.55	3.84	NO

### Putamen BOLD Response and Modality Overlap/Non-Overlap

**FIGURE 2:**
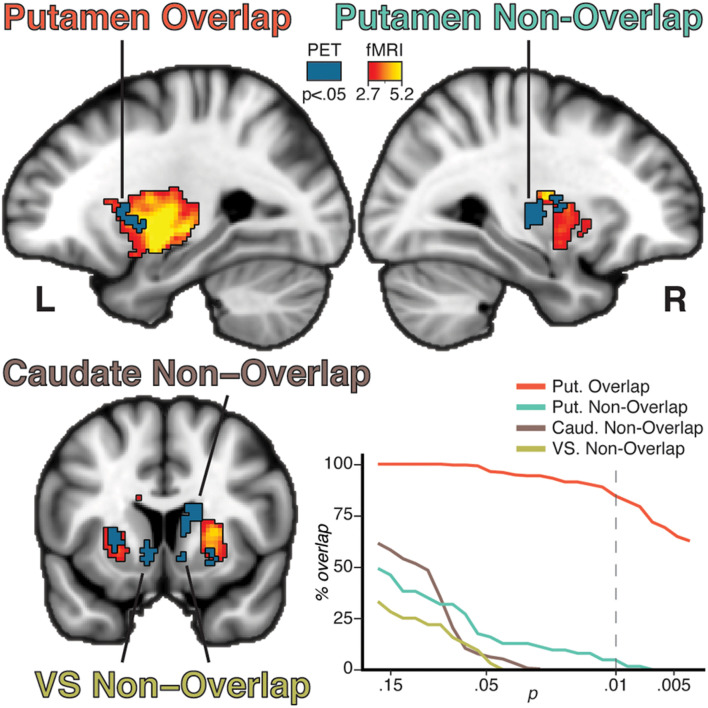
Results from the group lp-ntPET analysis (blue solid color) and the group analysis of the fMRI GLM (red to yellow gradient). Four unique patterns of clusters were observed based on modality overlap and anatomical belonging: putamen overlap, putamen non-overlap, caudate non-overlap, and VS non-overlap. Percentage overlap of the PET clusters was calculated for different fMRI thresholds (bottom right plot). Increasing the threshold reduced the percentage intersection of the non-overlapping clusters, while the overlapping clusters remain at a high level of intersection even at more conservative thresholds. Dotted line represents the threshold used in the brain figures.

The voxelwise fMRI GLM contrast [tapping > rest] showed a bilateral response in the putamen (left: *p* = 0.002, cluster size 990 voxels, peak t-stat = 8.06; right: *p* = 0.002, cluster size 953 voxels, peak t-stat = 5.25). The response was more wide-spread and stronger in the left putamen than in the right putamen (Figure 2), consistent with predominant contralateral activation during movement.

Overlap between [11C]Raclopride displacement and task fMRI response was observed in distinct parts of the putamen (Figure 2). The clusters overlapped well at all thresholds, with 84% of voxels overlapping at an fMRI threshold of *p* = 0.01. Conversely, the non-overlapping clusters showed poor overlap even at lower threshold, indicating that overlap/non-overlap definitions were relatively threshold-independent. Four general patterns were established: bilateral putamen overlap, ipsilateral putamen non-overlap, caudate non-overlap, and VS non-overlap (Figure 2). An exploratory analysis of the BOLD timecourse in the non-overlapping clusters showed that non-overlap was not driven by a shifted or negative BOLD signal in relation to the task regressor (Supplementary Figure 1, top).

A supplementary ROI based lp-ntPET analysis of the four clusters of interest confirmed [11C]Raclopride displacement for each ROI (Supplementary Figure 1, bottom). Moreover, this ROI-based lp-ntPET showed that differences in task block timing between individuals did not affect the ability to detect [11C]Raclopride displacement, supporting our approach that individual maps can be collapsed across subjects (Supplementary Material).

### Functional Coupling of Dopamine Release Clusters

**FIGURE 3:**
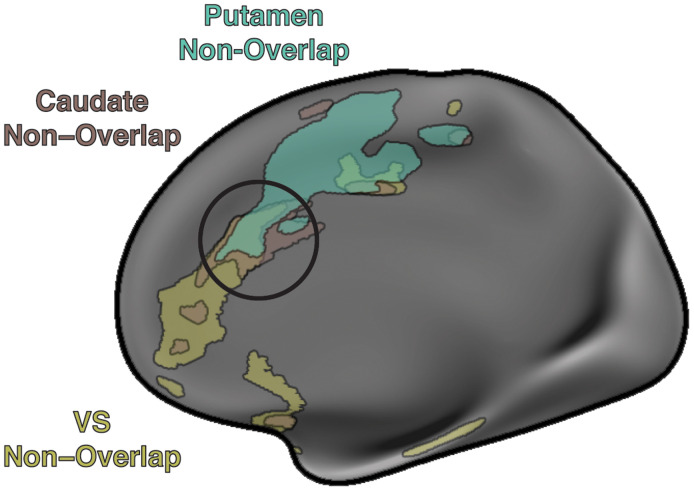
Cortical resting state functional coupling of the non-overlapping clusters. The putamen non-overlap cluster shows functional connectivity to the supplementary motor area and anterior cingulate cortex, the caudate non-overlap cluster shows functional connectivity to the anterior cingulate cortex, the VS non-overlap cluster show functional connectivity to the ventromedial prefrontal cortex and anterior cingulate cortex. A trilateral intersection of functional coupling is observed in the anterior cingulate cortex (black circle) possibly indicating a locus of integration among attentional (caudate), motivational (VS), and motor (putamen) component processes.

The voxelwise seed-based functional connectivity analysis showed that putamen overlap was functionally coupled to the bilateral motor cortices, supplementary motor area (SMA), anterior cingulate cortex (ACC), and insula. Putamen non-overlap showed similar functional coupling as the putamen overlap. Caudate non-overlap showed functional coupling to the ACC. VS non-overlap showed functional coupling to the medial prefrontal cortex and ACC (Supplementary Figure 2). Overlapping functional coupling between the putamen non-overlap, caudate non-overlap, and VS non-overlap seeds was observed in the ACC (Figure 3).

## Discussion

Striatal DA release is known to facilitate movements (Albin et al., 1989; DeLong, 1990; Calabresi et al., 2014). Previous studies have shown that unrewarded finger tapping elicits widespread striatal DA release (Badgaiyan et al., 2003; Goerendt et al., 2003) providing support for models that assert an important role for DA during motor function. In this study we utilized a novel multimodal PET and fMRI approach to provide support for the hypothesis that striatal DA release during finger tapping can be dissociated into both motor and non-motor component processes, which previously has not been possible with PET alone. Specifically, we exploited the temporal fidelity of fMRI to identify the spatial loci related to the direct motor component, while the PET measure of DA release was temporally insensitive thereby revealing the overall pattern of striatal DA during the task. The complementary information provided by both modalities permitted us to make conclusions about the overlapping and non-overlapping regions of responses. Overlap of responses was interpreted as DA signaling specifically related to motor function, while non-overlap was interpreted as DA related to the task as a whole which includes component processes related to attentional demands. Below, we discuss these findings in relation to existing knowledge on striatal functional organization.

### Dopamine Release and BOLD Response Overlap in the Bilateral Putamen During Finger Tapping

In line with previous comparisons across studies (Badgaiyan et al., 2003; Goerendt et al., 2003; Witt et al., 2008) and known anatomy, we observed overlapping loci of DA release and fMRI responses to finger tapping only in the putamen. As expected, this outcome concords with the putamen as the primary motor region of the striatum (Albin et al., 1989; DeLong, 1990; Calabresi et al., 2014), and provides direct evidence that fMRI responses in humans are spatially congruent with dopaminergic activity. Moreover, the location of overlapping BOLD response and DA release was functionally coupled in fMRI with the SMA and bilateral motor cortex (as well as ACC), providing further support that signals from both modalities reflect the modulation of activity in cortico-thalamic loops that regulate motor functions (Alexander et al., 1986). Because fMRI is cheaper, faster and less invasive than functional PET, this finding, albeit expected, highlights the potential for fMRI as a biomarker of dopaminergic function in the motor pathway, for example in studies of Parkinson’s disease (Bloem et al., 2019). The spatial convergence between sites of DA release and fMRI task activity encourages a larger study in which individual differences are probed to establish whether larger fMRI responses are proportional to greater DA release during a simple tapping task, which additionally would establish task fMRI as a biomarker of neurochemical dysfunction.

An intriguing and unexpected finding with respect to the motor compartment was that of DA release in the ipsilateral (right) posterior putamen, which was incongruent with the BOLD pattern. The lack of BOLD response in this area suggests a divergence of DA function compared to where the PET and fMRI modalities overlap, possibly relating to differences in DA release patterns (Liu et al., 2021) or differences of co-release of inhibitory, excitatory, or other modulatory neurotransmitters (Hnasko and Edwards, 2012; Tritsch et al., 2016). The posterior putamen was functionally coupled to similar areas as the overlapping putamen clusters (SMA, bilateral motor cortex, and ACC), suggesting a role in modulation of cortico-thalamic regulating motor functions. However, the exact nature of such modulation remains unclear. The putamen non-overlap cluster was uniquely identified by combining PET and fMRI imaging, which speaks to the value of multimodal imaging for researching novel biomarkers.

### Dopamine Release in the Caudate and Ventral Striatum Related to Finger Tapping

Striatal DA release in the caudate during finger tapping has been assumed to reflect learning, response selection, predictability of events, and progression tracking (Badgaiyan et al., 2003; Goerendt et al., 2003). Such interpretations have been made based on the locus of DA release, but it has not been possible to definitively dissociate this response from DA release concurrently observed in the putamen. The current task did not involve any apparent learning component nor complicated motor sequence executions, but it did involve predictable events. The BOLD response to event prediction could be expected to precede the event, causing a moderate fit to the defined regressor or even negatively correlating with the regressor. However, in an additional analysis, the observed BOLD response in caudate neither positively nor negatively correlated with the regressor (Supplementary Figure 1), which speaks against event prediction or other event-tied component process. An alternative possibility may then be a role in some form of sustained external or internal monitoring, which is common across different finger tapping tasks. The caudate is known to be related to executive functions such as generation and monitoring of appropriate strategies needed to achieve certain goals (Grahn et al., 2008). In support of this interpretation, the caudate DA release cluster was functionally coupled with the ACC (Figure 3). Among many functional roles assigned to the ACC, it is part of the brain’s attention network (Posner and Petersen, 1990; Weissman et al., 2005; Yeo et al., 2011), and has been associated with error monitoring (Kiehl et al., 2000; Swick and Turken, 2002). Thus, DA release in the caudate might be related to attentional demands associated with tracking the progression of a tapping sequence, consistent with the interpretation by Goerendt et al. (2003).

More generally, the present finding of dissociable PET-MRI pattern in putamen and caudate encourage the design of functional imaging experiments that are able to isolate component processes (e.g., by a mixed design that allows to model both sustained set and events). Such designs may then be able to identify and monitor regionally specific courses of DA degeneration in disease. To give a concrete example, in an fMRI-experiment the caudate response to task set may serve as a within-patient reference to infer the severity of Parkinson’s disease-pathology in the motor-putamen.

DA release was also observed in the bilateral VS, which to our knowledge has not been reported in the literature during unrewarded finger tapping which may reflect the fact that our PET model was less constrained than in previous work. The VS is an area integrally linked with incentive processing (e.g., Ikemoto and Panksepp, 1999; Knutson and Greer, 2008; Haber and Knutson, 2010; Sescousse et al., 2013; Salgado and Kaplitt, 2015; Wang et al., 2016; Grill et al., 2020), and VS DA release has been observed during rewarded conditions (Pappata et al., 2002; Joutsa et al., 2012; Jonasson et al., 2014). Recent findings have pointed to unidirectional open feedforward loops between the VS and sensorimotor areas of the striatum through the DAergic midbrain, through which the VS can influence selection and invigorate action based on emotional and motivational states (Aoki et al., 2019). The VS was most strongly functionally coupled to the vmPFC and ACC (Figure 3), replicating previous functional connectivity findings (Di Martino et al., 2008). The functional connectivity analysis of the striatal DA release clusters thus reveals trilateral functional coupling in the ACC (Figure 3), supporting the ACC being viewed as an integrative area for component processes related to motivation, attention, and motor control (Paus et al., 2001).

In conclusion, we could separate motor from non-motor component processes of finger tapping based on the complementary information from PET and fMRI, but the current experimental design did not support strong conclusions as to what the non-motor components represent. Thus, what is interpreted as non-motor components may reflect sustained executive components of the task or they may be essential parts of finger tapping, for example involving aspects of rhythm or timing of the current task structure.

### Methodological Advances

An exciting avenue of research has opened up with hybrid PET/MR systems allowing the investigation of concomitant neurochemical and vascular changes in response to various stimuli. Advances in functional PET analysis (lp-ntPET; Normandin et al., 2012; Sander et al., 2013; Johansson et al., 2019) have made it possible to more accurately model onset and durations of experimental manipulations, thereby allowing for more flexible and “fMRI like” PET paradigms. In our main results we use lp-ntPET to voxelwise identify spatial regions indicating [11C]Raclopride displacement. The lp-ntPET method is also able to dynamically estimate binding potentials during the task which can be translated into DA D2 occupancy in the case of [11C]Raclopride (Supplementary Figure 1). Future studies with a larger sample could potentially characterize regional and inter-individual differences in occupancy functions associated with traits and/or behavioral manipulations related to motor, executive, and/or incentive processing. In this study, we pushed the temporal limits of lp-ntPET utilizing various onsets, durations, and number of experimental manipulation blocks. For our main results, it was possible to collapse spatial maps across participants and task paradigms since we were investigating voxels that exhibited DA release. Further methodological considerations are needed when interpreting occupancy functions of more than a single task block.

### Limitations

There are several limitations to consider in this study. The study has low power compared to contemporary neuro imaging studies, and larger-scale studies are called for. With this caveat we note that previous PET studies investigating striatal DA release in relation to finger tapping have shown a robust effect at the single subject level (Badgaiyan et al., 2003) as well as stable group effects for small samples (Goerendt et al., 2003), and robust single-subject BOLD responses have been observed in fMRI studies (Moritz et al., 2000).

The logic of interpreting spatial overlap/non-overlap between [11C]Raclopride displacement clusters and concomitant BOLD response also has its limitations. For overlapping modalities to be related to each other it assumes a neurovascular coupling between DA and the BOLD response. Neurovascular coupling of DA receptors has been observed (Choi et al., 2006; Knutson and Gibbs, 2007; Sander et al., 2013) but the exact nature of the coupling and how it impacts the BOLD response is still unclear. Also, if modality overlap represents signals related to each other, a closer peak-to-peak overlap could be expected. Even if both modalities are collected at the same time, spatial resolution and field of view between modalities differ, possibly causing discrepancies when normalizing to a common template. The stepwise percentage overlap evaluation did nevertheless support the overlap/non-overlap definitions.

Finally, DA release in [11C]Raclopride displacement clusters not accompanied by a BOLD response may still impact the BOLD signal but at a time scale not detectable by the *a priori* defined fMRI GLM regressor. Unfortunately, the current task design did not lend itself to be analyzed for neither more sustained BOLD response (due to the exceedingly long task blocks) nor faster BOLD response (due to TR limitations). Similarly, the current task design did not lend itself for finer control of the relation between finger tapping speed and dopamine release since we let participants determine their own speed. Controlling tapping frequency across individuals may be an improvement over the current design. Alternatively, using similar methods as described here, a better powered study could potentially investigate individual differences of self-paced tapping frequency and striatal DA response. To substantiate our interpretations of component processes related to DA release clusters, we utilized seed-based resting state functional connectivity. This method has previously been used to identify brain networks associated with a seed (Di Martino et al., 2008). Here, we use it as a proxy for function which should not be taken as a definitive component process description, but rather as an indication of functional role.

## Conclusion

In this study we explore DA release patterns during an unrewarded finger tapping task using a novel hybrid PET-fMRI imaging approach. DA release in bilateral putamen spatially overlapped with concomitant BOLD response. This finding highlights the potential for fMRI as a biomarker of dopaminergic function in the motor pathway, for example in studies of Parkinson’s disease. We also observed DA release that did not overlap with the striatal BOLD response in caudate and VS, indicating component processes of finger tapping that are reliant on DA but unrelated to motor action. The non-overlapping areas showed distinct functional connectivity profiles that intersected in the ACC, supporting the view of ACC as an integrative area for component processes related to motivation, attention, and motor control.

## Data Availability Statement

Neuroimaging data on the group level can be viewed and downloaded from https://neurovault.org/collections/11584/.

## Ethics Statement

The studies involving human participants were reviewed and approved by the Regional Ethics Committee at Umeå University. The patients/participants provided their written informed consent to participate in this study.

## Author Contributions

FG conducted the final analysis and wrote the first draft. JJ conducted analysis and edited the manuscript. JA developed the PET protocol and edited the manuscript. PB developed the MR protocol and edited the manuscript. LN conceived the study and edited the manuscript. AR conceived the study, conducted analysis, and edited the manuscript. All authors contributed to the article and approved the submitted version.

## Conflict of Interest

The authors declare that the research was conducted in the absence of any commercial or financial relationships that could be construed as a potential conflict of interest.

## Publisher’s Note

All claims expressed in this article are solely those of the authors and do not necessarily represent those of their affiliated organizations, or those of the publisher, the editors and the reviewers. Any product that may be evaluated in this article, or claim that may be made by its manufacturer, is not guaranteed or endorsed by the publisher.

## Footnotes

1
https://fsl.fmrib.ox.ac.uk/

## References

[B1] Albin R. L. Young A. B. Penny J. B. (1989). The functional anatomy of basal ganglia disorders. *Trends Neurosci.* 12 366–375. 10.1016/0166-2236(89)90074-X 2479133

[B2] Alexander G. DeLong M. R. Strick P. L. (1986). Parallel Organization of Functionally Segregated Circuits Linking Basal Ganglia and Cortex. *Annu. Rev. Neurosci.* 9 357–381. 10.1146/annurev.neuro.9.1.357 3085570

[B3] Aoki S. Smith J. B. Li H. Yan X. Igarashi M. Coulon P. (2019). An open cortico-basal ganglia loop allows limbic control over motor output via the nigrothalamic pathway. *eLife* 8, 1–29. 10.7554/eLife.49995 31490123 PMC6731092

[B4] Badgaiyan R. D. Fischman A. J. Alpert N. M. (2003). Striatal dopamine release during unrewarded motor task in human volunteers. *NeuroReport* 14 1421–1424. 10.1097/00001756-200308060-00003 12960756

[B5] Belujon P. Grace A. A. (2017). Dopamine system dysregulation in major depressive disorders. *Int. J. Neuropsychopharmacol.* 20 1036–1046. 10.1093/ijnp/pyx056 29106542 PMC5716179

[B6] Bloem B. R. Marks W. J. Silva, De Lima A. L. Kuijf M. L. Van Laar T. (2019). The Personalized Parkinson Project: Examining disease progression through broad biomarkers in early Parkinson’s disease. *BMC Neurol.* 19:1–10. 10.1186/s12883-019-1394-3 31315608 PMC6636112

[B7] Brisch R. Saniotis A. Wolf R. Bielau H. Bernstein H. G. Steiner J. (2014). The role of dopamine in schizophrenia from a neurobiological and evolutionary perspective: Old fashioned, but still in vogue. *Front. Psychiatry* 5:1–11. 10.3389/fpsyt.2014.00047 24904434 PMC4032934

[B8] Calabresi P. Picconi B. Tozzi A. Ghiglieri V. Di Filippo M. (2014). Direct and indirect pathways of basal ganglia: A critical reappraisal. *Nat. Neurosci.* 17 1022–1030. 10.1038/nn.3743 25065439

[B9] Choi J. K. Chen Y. I. Hamel E. Jenkins B. G. (2006). Brain hemodynamic changes mediated by dopamine receptors: Role of the cerebral microvasculature in dopamine-mediated neurovascular coupling. *NeuroImage* 30 700–712. 10.1016/j.neuroimage.2005.10.029 16459104

[B10] Christian B. T. Vandehey N. T. Floberg J. M. Mistretta C. A. (2010). Dynamic PET denoising with HYPR processing. *J. Nucl. Med.* 51, 1147–1154. 10.2967/jnumed.109.073999 20554743 PMC3250311

[B11] Cohen M. X. Frank M. J. (2009). Neurocomputational models of basal ganglia function in learning, memory and choice. *Behav. Brain Res.* 199 141–156. 10.1016/j.bbr.2008.09.029 18950662 PMC2762323

[B12] Dahlin E. Stigsdotter Neely A. Larsson A. Bäckman L. Nyberg L. (2008). Transfer of Learning After Updating Training Mediated by the Striatum. *Science* 320 1510–1512. 10.1126/science.1155466 18556560

[B13] DeLong M. R. (1990). Primate models of movement disorders of basal ganglia origin. *Trends Neurosci.* 13 281–285. 10.1016/0166-2236(90)90110-V 1695404

[B14] Dewey S. L. Smith G. S. Logan J. Brodie J. D. Fowler J. S. Wolf A. P. (1993). Striatal binding of the PET ligand 11C-raclopride is altered by drugs that modify synaptic dopamine levels. *Synapse* 13 350–356. 10.1002/syn.890130407 8480281

[B15] Di Martino A. Scheres A. Margulies D. S. Kelly A. M. C. Uddin L. Q. Shehzad Z. (2008). Functional Connectivity of Human Striatum: A Resting State fMRI Study. *Cereb. Cortex* 18 2735–2747. 10.1093/cercor/bhn041 18400794

[B16] Elsinger C. L. Rao S. M. Zimbelman J. L. Reynolds N. C. Blindauer K. A. Hoffmann R. G. (2003). Neural basis for impaired time reproduction in Parkinson’s disease: An fMRI study. *J. Int. Neuropsychol. Soc.* 9 1088–1098. 10.1017/S1355617703970123 14738289

[B17] Farde L. Hall H. Ehrin E. Sedvall G. (1986). Quantitative analysis of D2 dopamie receptor binding in the living human brain by PET. *Science* 231 258–261. 10.1126/science.2867601 2867601

[B18] Fischl B. (2012). FreeSurfer. *NeuroImage* 62 774–781. 10.1016/j.neuroimage.2012.01.021 22248573 PMC3685476

[B19] Goerendt I. K. Messa C. Lawrence A. D. Grasby P. M. Piccini P. Brooks D. J. (2003). Dopamine release during sequential finger movements in health and Parkinson’s disease: A PET study. *Brain* 126 312–325. 10.1093/brain/awg035 12538400

[B20] Grahn J. A. Parkinson J. A. Owen A. M. (2008). The cognitive functions of the caudate nucleus. *Prog. Neurobiol.* 86 141–155. 10.1016/j.pneurobio.2008.09.004 18824075

[B21] Grill F. Nyberg L. Rieckmann A. (2020). Neural correlates of reward processing: Functional dissociation of two components within the ventral striatum. *Brain Behav.* 11 1–12. 10.1002/brb3.1987 33300306 PMC7882172

[B22] Haber S. N. Knutson B. (2010). The reward circuit: Linking primate anatomy and human imaging. *Neuropsychopharmacology* 35 4–26. 10.1038/npp.2009.129 19812543 PMC3055449

[B23] Hnasko T. S. Edwards R. H. (2012). Neurotransmitter corelease: mechanism and physiological role. *Annu. Rev. Physiol.* 74, 225–243. 10.1146/annurev-physiol-020911-153315 22054239 PMC4090038

[B24] Ikemoto S. Panksepp J. (1999). The role of nucleus accumbens dopamine in motivated behavior: A unifying interpretation with special reference to reward-seeking. *Brain Res. Rev.* 31 6–41. 10.1016/S0165-0173(99)00023-5 10611493

[B25] Johansson J. Hirvonen J. Lovró Z. Ekblad L. Kaasinen V. Rajasilta O. (2019). Intranasal naloxone rapidly occupies brain mu-opioid receptors in human subjects. *Neuropsychopharmacology* 44 1667–1673. 10.1038/s41386-019-0368-x 30867551 PMC6785104

[B26] Jonasson L. S. Axelsson J. Riklund K. Braver T. S. Ögren M. Bäckman L. (2014). Dopamine release in nucleus accumbens during rewarded task switching measured by [11C]raclopride. *NeuroImage* 99 357–364. 10.1016/j.neuroimage.2014.05.047 24862078

[B27] Joutsa J. Johansson J. Niemelä S. Ollikainen A. Hirvonen M. M. Piepponen P. (2012). Mesolimbic dopamine release is linked to symptom severity in pathological gambling. *NeuroImage* 60 1992–1999. 10.1016/j.neuroimage.2012.02.006 22348881

[B28] Kiehl K. A. Liddle P. F. Hopfinger J. B. (2000). Error processing and the rostral anterior cingulate: An event-related fMRI study. *Psychophysiology* 37 216–223. 10.1017/S0048577200990231 10731771

[B29] Knutson B. Gibbs S. E. B. (2007). Linking nucleus accumbens dopamine and blood oxygenation. *Psychopharmacology* 191 813–822. 10.1007/s00213-006-0686-7 17279377

[B30] Knutson B. Greer S. M. (2008). Anticipatory affect: neural correlates and consequences for choice. *Philos. Trans. R. Soc. Lond. B Biol. Sci.* 363, 3771–3786. 10.1098/rstb.2008.0155 18829428 PMC2607363

[B31] Lappin J. M. Reeves S. J. Mehta M. A. Egerton A. Coulson M. Grasby P. M. (2009). Dopamine release in the human striatum: Motor and cognitive tasks revisited. *J. Cereb. Blood Flow Metabol.* 29 554–564. 10.1038/jcbfm.2008.146 19088741

[B32] Laruelle M. (2000). Imaging synaptic neurotransmission with in vivo binding competition techniques: a critical review. *J. Cereb. Blood Flow Metab.* 20, 423–451. 10.1097/00004647-200003000-00001 10724107

[B33] Lehéricy S. Bardinet E. Tremblay L. Van De Moortele P. F. Pochon J. B. Dormont D. (2006). Motor control in basal ganglia circuits using fMRI and brain atlas approaches. *Cereb. Cortex* 16 149–161. 10.1093/cercor/bhi089 15858164

[B34] Liu C. Goel P. Kaeser P. S. (2021). Spatial and temporal scales of dopamine transmission. *Nat. Rev. Neurosci.* 22 345–358. 10.1038/s41583-021-00455-7 33837376 PMC8220193

[B35] Madsen M. T. (1992). A simplified formulation of the gamma variate function. *Physics Med. Biol.* 37 1597–1600. 10.1088/0031-9155/37/7/010

[B36] Martini A. Dal Lago D. Edelstyn N. M. J. Salgarello M. Lugoboni F. Tamburin S. (2018). Dopaminergic Neurotransmission in Patients With Parkinson’s Disease and Impulse Control Disorders: A Systematic Review and Meta-Analysis of PET and SPECT Studies. *Front. Neurol.* 9:01018. 10.3389/fneur.2018.01018 30568628 PMC6290338

[B37] Monchi O. Ko J. H. Strafella A. P. (2006). Striatal dopamine release during performance of executive functions: A [11C] raclopride PET study. *NeuroImage* 33 907–912. 16982202 10.1016/j.neuroimage.2006.06.058 PMC2967527

[B38] Moritz C. H. Haughton V. M. Cordes D. Quigley M. Meyerand M. E. (2000). Whole-brain functional MR imaging activation from a finger-tapping task examined with independent component analysis. *AJNR Am. J. Neuroradiol.* 21, 1629–1635. 11039341 PMC8174873

[B39] Nevalainen N. Riklund K. Andersson M. Axelsson J. Ögren M. Lövdén M. (2015). COBRA: A prospective multimodal imaging study of dopamine, brain structure and function, and cognition. *Brain Res.* 1612 83–103. 10.1016/j.brainres.2014.09.010 25239478

[B40] Normandin M. D. Schiffer W. K. Morris E. D. (2012). A linear model for estimation of neurotransmitter response profiles from dynamic PET data. *NeuroImage* 59 2689–2699. 10.1016/j.neuroimage.2011.07.002 21767654 PMC3702051

[B41] Pappata S. Dehaene S. Poline J. B. Gregoire M. C. Jobert A. Delforge J. (2002). In Vivo detection of striatal dopamine release during reward: A PET study with [11C]raclopride and a single dynamic scan approach. *NeuroImage* 16 1015–1027. 10.1006/nimg.2002.1121 12202089

[B42] Paus T. (2001). Primate anterior cingulate cortex: where motor control, drive and cognition interface. *Nat. Rev. Neurosci.* 2, 417–424. 10.1038/35077500 11389475

[B43] Petersen S. E. Dubis J. W. (2012). The mixed block/event-related design. *NeuroImage* 62 1177–1184. 10.1016/j.neuroimage.2011.09.084 22008373 PMC3288695

[B44] Posner M. I. Petersen S. E. (1990). The attention system of the human brain. *Annu. Rev. Neurosci.* 13 25–42. 10.1146/annurev.ne.13.030190.000325 2183676

[B45] Riecker A. Wildgruber D. Mathiak K. Grodd W. Ackermann H. (2003). Parametric analysis of rate-dependent hemodynamic response functions of cortical and subcortical brain structures during auditorily cued finger tapping: A fMRI study. *NeuroImage* 18 731–739. 10.1016/S1053-8119(03)00003-X 12667850

[B46] Salgado S. Kaplitt M. G. (2015). The nucleus accumbens: A comprehensive review. *Stereotactic Funct. Neurosurg.* 93 75–93. 10.1159/000368279 25720819

[B47] Sander C. Y. Hooker J. M. Catana C. Normandin M. D. Alpert N. M. Knudsen G. M. (2013). Neurovascular coupling to D2/D3 dopamine receptor occupancy using simultaneous PET/functional MRI. *Proc. Natl. Acad. Sci. U S A.* 110 11169–11174. 10.1073/pnas.1220512110 23723346 PMC3703969

[B48] Sescousse G. Caldú X. Segura B. Dreher J. C. (2013). Processing of primary and secondary rewards: A quantitative meta-analysis and review of human functional neuroimaging studies. *Neurosci. Biobehav. Rev.* 37 681–696. 10.1016/j.neubiorev.2013.02.002 23415703

[B49] Swick D. Turken U. (2002). Dissociation between conflict detection and error monitoring in the human anterior cingulate cortex. *Proc. Natl. Acad. Sci. U. S. A.* 99 16354–16359. 10.1073/pnas.252521499 12456882 PMC138615

[B50] Tritsch N. X. Granger A. J. Sabatini B. L. (2016). Mechanisms and functions of GABA co-release. *Nat. Rev. Neurosci.* 17 139–145. 10.1038/nrn.2015.21 26865019 PMC6980171

[B51] Turesky T. K. Olulade O. A. Luetje M. M. Eden G. F. (2018). An fMRI study of finger tapping in children and adults. *Hum. Brain Mapp.* 39 3203–3215. 10.1002/hbm.24070 29611256 PMC6052794

[B52] Wang K. S. Smith D. V. Delgado M. R. (2016). Using fMRI to study reward processing in humans: past, present, and future. *J. Neurophysiol.* 115 1664–1678. 10.1152/jn.00333.2015 26740530 PMC4808130

[B53] Watabe H. Endres C. J. Breier A. Schmall B. Eckelman W. C. Carson R. E. (2000). Measurement of dopamine release with continuous infusion of [11C]raclopride: optimization and signal-to-noise considerations. *J. Nucl. Med.* 41, 522–530. 10716328

[B54] Weissman D. H. Gopalakrishnan A. Hazlett C. J. Woldorff M. G. (2005). Dorsal anterior cingulate cortex resolves conflict from distracting stimuli by boosting attention toward relevant events. *Cereb. Cortex* 15 229–237. 10.1093/cercor/bhh125 15238434

[B55] Witt S. T. Laird A. R. Meyerand M. E. (2008). Functional neuroimaging correlates of finger-tapping task variations: An ALE meta-analysis. *NeuroImage* 42 343–356. 10.1016/j.neuroimage.2008.04.025 18511305 PMC2592684

[B56] Wu C. C. Fairhall S. L. McNair N. A. Hamm J. P. Kirk I. J. Cunnington R. (2010). Impaired sensorimotor integration in focal hand dystonia patients in the absence of symptoms. *J. Neurol. Neurosurg. Psychiatry* 81 659–665. 10.1136/jnnp.2009.185637 19965853

[B57] Wurster C. D. Graf H. Ackermann H. Groth K. Kassubek J. Riecker A. (2015). Neural correlates of rate-dependent finger-tapping in Parkinson’s disease. *Brain Struct. Funct.* 220 1637–1648. 10.1007/s00429-014-0749-1 24647755

[B58] Yeo B. T. T. Krienen F. M. Sepulcre J. Sabuncu M. R. Lashkari D. Hollinshead M. (2011). The organization of the human cerebral cortex estimated by intrinsic functional connectivity. *J. Neurophysiol.* 106 2322–2345. 10.1152/jn.00339.2011 21653723 PMC3174820

