# Supplementary material for: Dissecting Motor and Cognitive Component Processes of a Finger-Tapping Task With Hybrid Dopamine Positron Emission Tomography and Functional Magnetic Resonance Imaging
Source: Front Hum Neurosci. 2021 Nov 29;15:733091. doi: 10.3389/fnhum.2021.733091 (PMC8667474; doi:10.3389/fnhum.2021.733091)

Supplementary Material

As expected, the BOLD signal in putamen overlap ROI matches well with the onset and offset of tapping within the task blocks while the other ROIs fit less well (putamen overlap: $r = .44$, $SD = .23$; putamen non-overlap: $r = .17$, $SD = .14$; caudate non-overlap: $r = .02$, $SD = .22$; VS non-overlap: $r = .08$, $SD = .22$; Figure S1 top).

An additional lp-ntPET analysis was performed on putamen overlap, putamen non-overlap, caudate non-overlap, and VS non-overlap ROIs to calculate dynamic dopamine occupancy curves for which the peak occupancy were extracted (Putamen Overlap: Mean = 16.70%, SD = 10.51%; Putamen non-overlap: M = 37.78%, SD = 13.93%; Caudate non-overlap: M = 22.03%, SD = 5.63%; VS non-overlap: M = 28.10%, SD = 13.07%) The ANOVA on the peak occupancy showed a significant effect of ROI ($F(3,24) = 7.55$, $p < .001$), post-hoc paired sample t-tests confirmed a significant difference between putamen non-overlap and all other ROIs (Shapiro-Wilk normality test all non-significant). The occupancy curves confirmed [11C]Raclopride displacement for all ROIs and task complexities which, through visual inspection were congruent with task block onsets (Figure S1 bottom).

Figure S1. Top: mean BOLD response for the various clusters over three minutes of the blocks. As expected, the BOLD signal in putamen overlap ROI matches well with the onset and offset of tapping within the task blocks while the other ROIs fit less well. Bottom: mean percentage occupancy calculations from the dynamic occupancy curves for the different task versions (task A and B (Figure 1) averaged to single block task). The putamen non-overlap occupancy curve has a significant higher peak than all other curves. However, these results should be interpreted with caution. The occupancy curves fit well with block onsets as well as confirming DA release in all separate clusters.

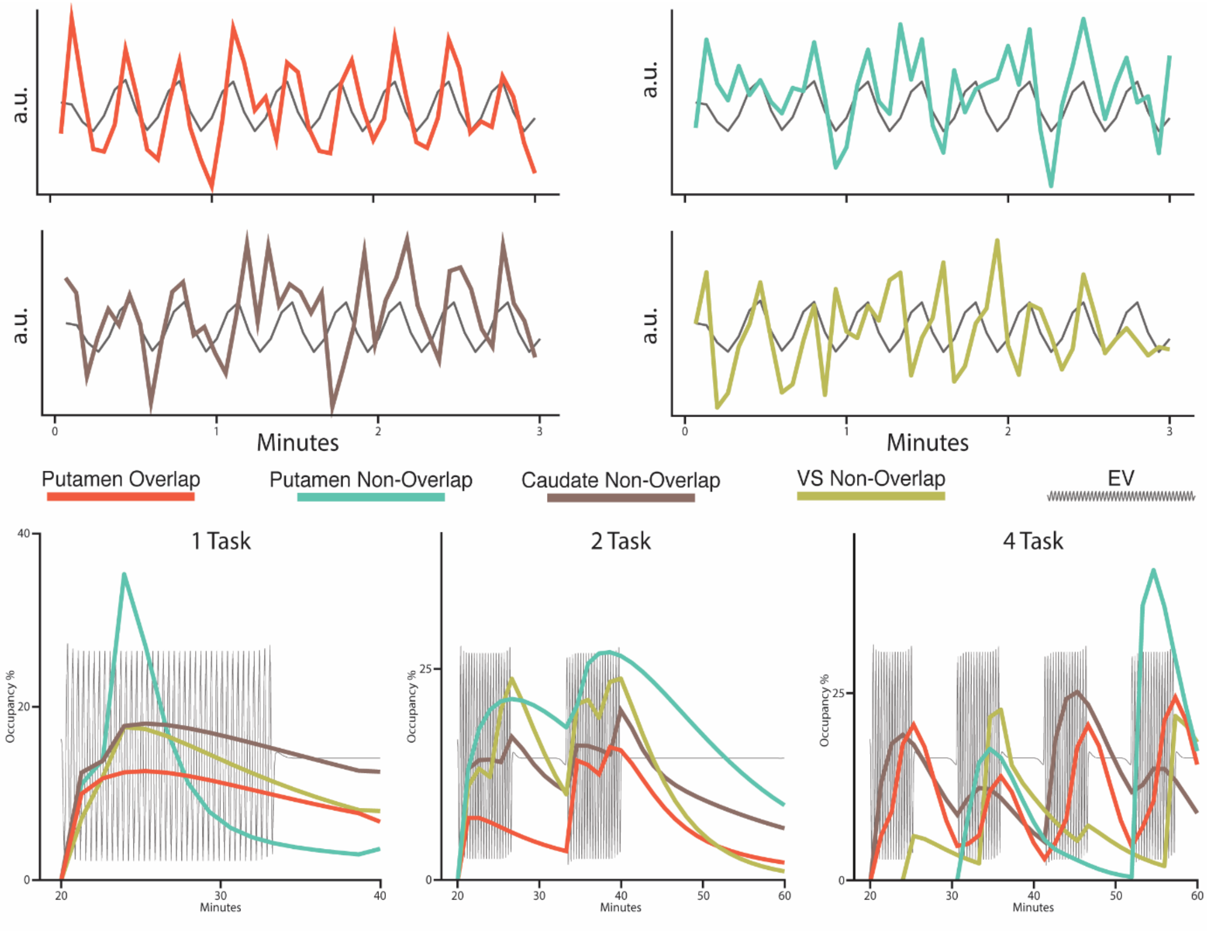

Figure S2. Cortical functional coupling of all PET clusters separately. Color bar represents t-statistics.

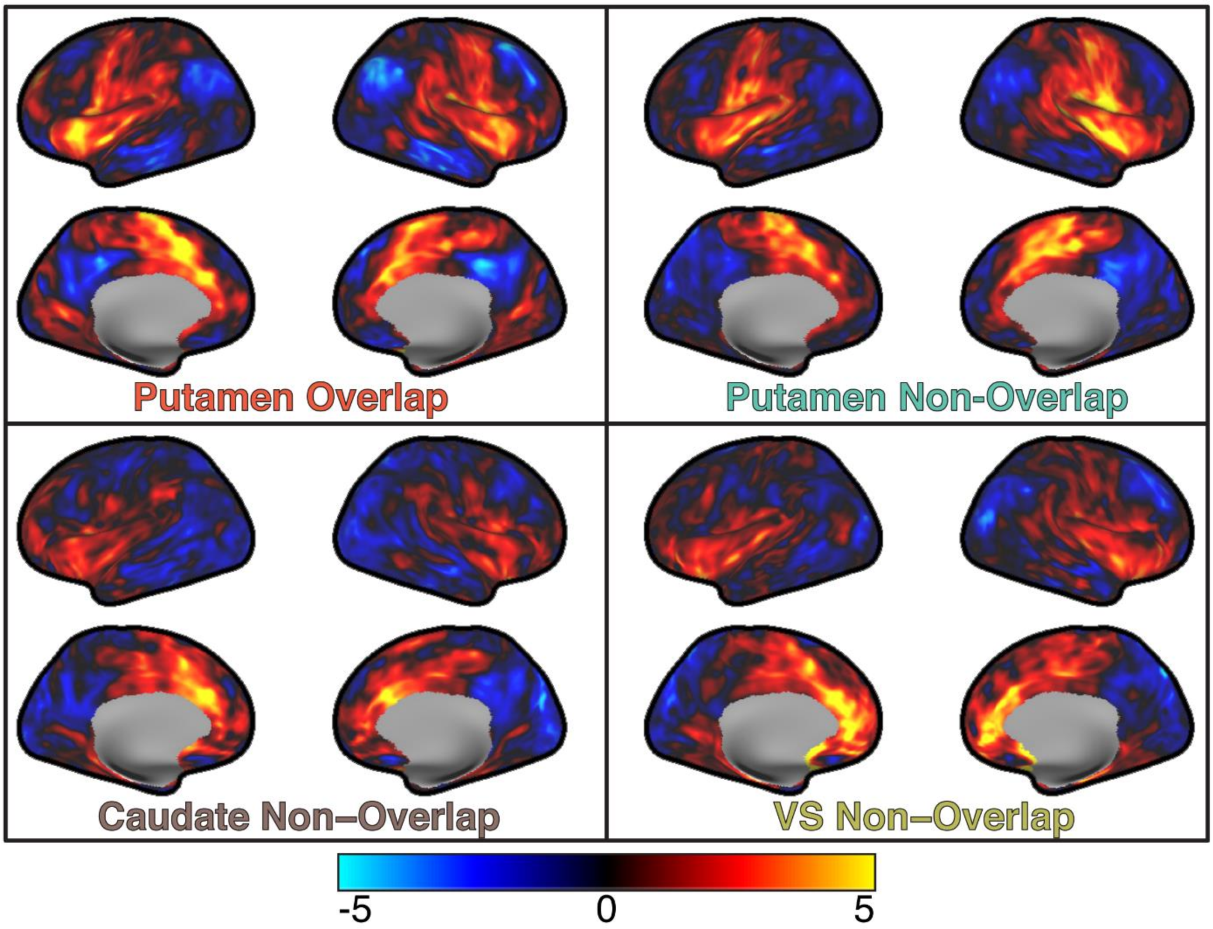

Supplement: Supplementary file 1 [file Data_Sheet_1.pdf]
